# Supplementary material for: Evidence From Web-Based Dietary Search Patterns to the Role of B12 Deficiency in Non-Specific Chronic Pain: A Large-Scale Observational Study
Source: J Med Internet Res. 2018 Jan 5;20(1):e4. doi: 10.2196/jmir.8667 (PMC5775484; doi:10.2196/jmir.8667)
Supplement: Multimedia Appendix 1 [file jmir_v20i1e4_app1.pdf]

Terms used in the study

Abilify

Acid Gone

Aciphex

AcipHex Sprinkle

Alamag

Alamag Plus

Alcalak

alginic acid

Alka-Mints

Almacone

Alternagel

aluminum hydroxide

Ami-Lac

Amitriptyline

Anafranil

aripiprazole

Atypical antidepressants

Atypical antidepressants

Axid

Barrett's esophagus

brexpiprazole

Bright-light therapy

Brintellix

bupropion

Calcium Channel Alpha-2-delta Ligands

Calcium Oyster Shell

Cal-Gest

Caltrate

Celexa

Cimetidine

Citalopram

Clomipramine

CRPS

Cymbalta

Desipramine

desvenlafaxine

Desyrel

Dexilant

Dexlansoprazole

Di-Gel aluminum hydroxide

Doxepin

Dulcolax Milk of Magnesia

Duloxetine

Duloxetine

Effexor

Elavil

Electroconvulsive therapy

Emsam

endoscopy

epigastric pain

Escitalopram

Esomeprazole

esophagitis

Ex-Lax Milk of Magnesia

Famotidine

Fetzima

Fibromyalgia

Fluoxetine

Fluvoxamine

Gabapentin

Gastrinoma

gastritis

Gastroesophageal reflux disease

gastroscopy

Gaviscon Extra Strength

Gaviscon Regular Strength Liquid

Gaviscon Regular Strength Tablets

Gaviscon-2

Gelusil

Genaton

GERD

GORD

H2 antagonists

H2 receptor antagonists

heartburn

Helicobacter pylori

Icar Prenatal Chewable Calcium

Imipramine

isocarboxazid

Kapidex

Lansoprazole

lanton

levomilnacipran

levorphanol

Lexapro

Losec

Luvox

Lyrica

Maalox Advanced Regular Strength

Maalox Regular Strength

magaldrate

magnesium carbonate

magnesium hydroxide

magnesium trisilicate

magnesium trisilicate

Marplan

Masanti Supreme

medical cannabis

methadone

Mi-Acid

Mi-Acid Double Strength

Milantex

Milk of Magnesia

Milnaciprin

Mintox

Mintox Plus

mirtazapine

Monoamine oxidase inhibitors

morphine

Mylanta

Mylanta Gelcap

Mylanta Maximum Strength

Mylanta Supreme

Mylanta Tonight

Mylanta Ultra

Nardil

nefazodone

Neurontin

neuropathic pain

neuropathy

Neut

Neut Pro

Nexium

Nexum

nizatidine

Norpramin

Nortriptyline

NSAIDs

Omeprazole

Opiates

Os-Cal

oxycodone

Oysco 500

Oyster Calcium

Oyster Shell Calcium 500

Pamelor

Pantoprazole

Paroxetine

Paxil

Pedia-Lax Chewable Tablets

Pepcid

peptic ulcer disease

Pepto Children

phenelzine

Phillips Milk of Magnesia

Pregabalin

Prevacid

Prilosec

Pristiq

proton pump inhibitor

Protonix

Protriptyline

Prozac

PUD

Rabeprazole

Ranitidine

reflux

Remeron

Rexulti

Ri-Mag magaldrate

Riopan Plus magaldrate

Rolaids Extra Strength

Rolaids Extra Strength Softchews

Rolaids Regular Strength

Ron Acid

Ron-Acid Plus

Rulox

Savella

SDAM

seasonal affective disorder

Selective serotonin reuptake inhibitors

selegiline

Serotonin/norepinephrine reuptake inhibitors

Serotonin-Dopamine Activity Modulators

Serotonin-norepinephrine Reuptake Inhibitors

Sertraline

simethicone

simethicone

Sinequan

SNRIs

sodium bicarbonate

SSRIs

St. John's wort

st johns wort

stress ulcer

Surmontil

Tagamet

Tazac

Titralac calcium carbonate

Titralac Plus

Tofranil

Topical Lidocaine

Tramadol

Transcranial magnetic stimulation

tranylcypromine

trazodone

Tricyclic antidepressants

Trimipramine

Tums Kids

Tums Plus

Tums Regular Strength

Ultram

venlafaxine

Viibryd

Vilazodone

Vivactil

Vortioxetine

Wellbutrin

widespread pain

Zantac

Zollinger-Ellison syndrome

Zoloft
