# Supplementary material for: Evidence From Web-Based Dietary Search Patterns to the Role of B12 Deficiency in Non-Specific Chronic Pain: A Large-Scale Observational Study
Source: J Med Internet Res. 2018 Jan 5;20(1):e4. doi: 10.2196/jmir.8667 (PMC5775484; doi:10.2196/jmir.8667)
Supplement: Multimedia Appendix 2 [file jmir_v20i1e4_app2.pdf]

Appendix 2: Demographics

We assessed the characteristics of the population which searches for recipes by analyzing behavioral data collected from an opt-in consumer panel recruited by Internet analytics company comScore. Millions of panelists provide comScore with explicit permission to passively measure their online activities using monitoring software installed on their computers. In addition to logged search behavior, the comScore data also provides us with panelists' gender and age (mostly bucketed in 5-year increments).

Figure 1 shows the fraction of people making recipes searches by gender and age group. As this figure shows, older people query more for recipes, though the differences (excluding the 13-14 year olds) are small. The differences between males and females are even smaller, with the ratio of females to males searching for recipes lying in the range of 98% to 115%.

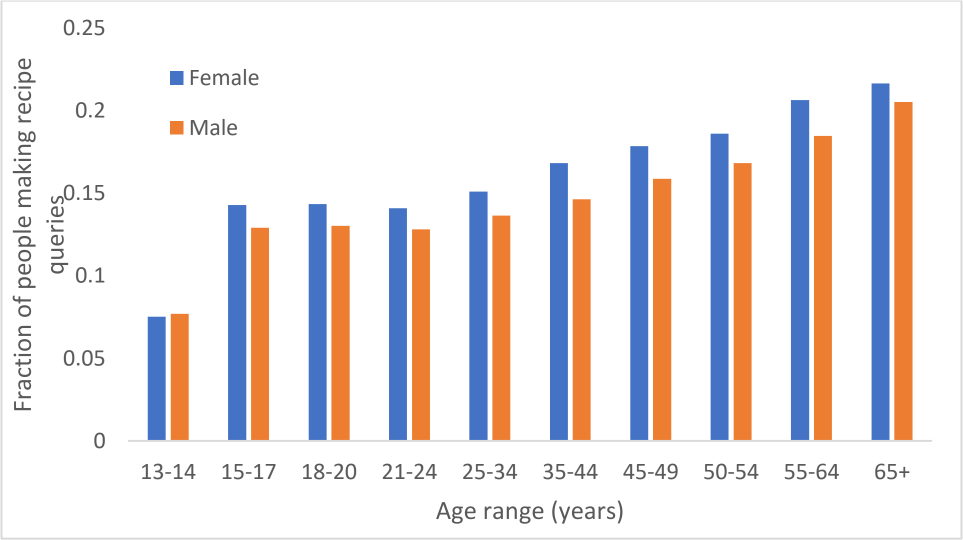

Figure 1: Fraction of people making recipe searches by females and by males, stratified by age.

Figure 2 shows the fraction of people searching for recipes as a function of income and of education. Here higher income is associated with a higher percentage of searches for recipes as is higher education, though as above, the differences are small.

We conclude that people who search for recipes are, in general, older and wealthier, though the differences in use are small. Thus, our dataset is representative of Internet users in the US.

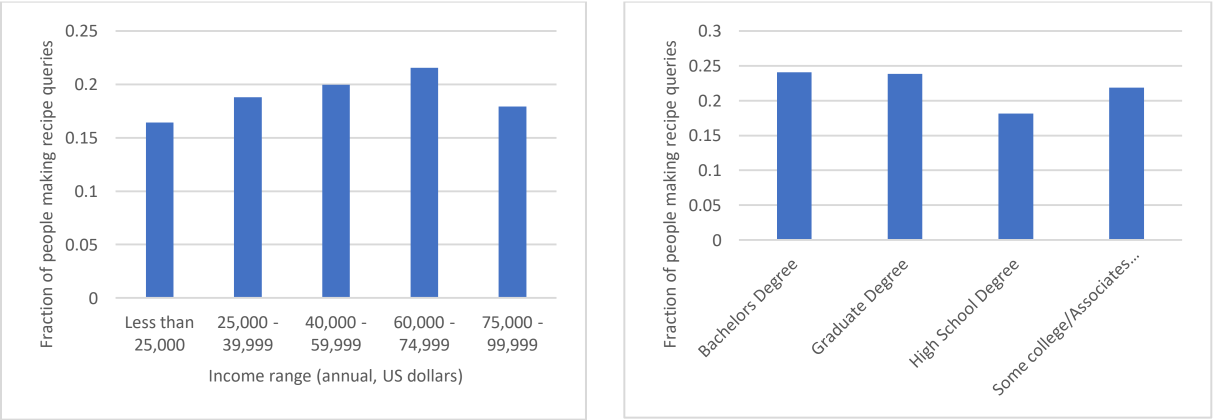

Figure 2: Fraction of people searching for recipes as a function of income (left) and education (right).

Finally, we modelled the estimated B12 consumption as a function of age and gender using a linear model. The R^2 of the model was very low, at 0.0007 ($P<10^{-10}$, N=387644). The coefficient for age was estimated at 0.007, meaning that B12 consumption was slightly higher for older people. The coefficient for gender was estimated at -0.15, meaning that females consume foods slightly richer in B12. However, both these effects are very small, indicating similar estimated consumption across ages and genders.
