# Supplementary material for: Evidence From Web-Based Dietary Search Patterns to the Role of B12 Deficiency in Non-Specific Chronic Pain: A Large-Scale Observational Study
Source: J Med Internet Res. 2018 Jan 5;20(1):e4. doi: 10.2196/jmir.8667 (PMC5775484; doi:10.2196/jmir.8667)
Supplement: Multimedia Appendix 3 [file jmir_v20i1e4_app3.pdf]

Multimedia Appendix 3: Maps of food consumption

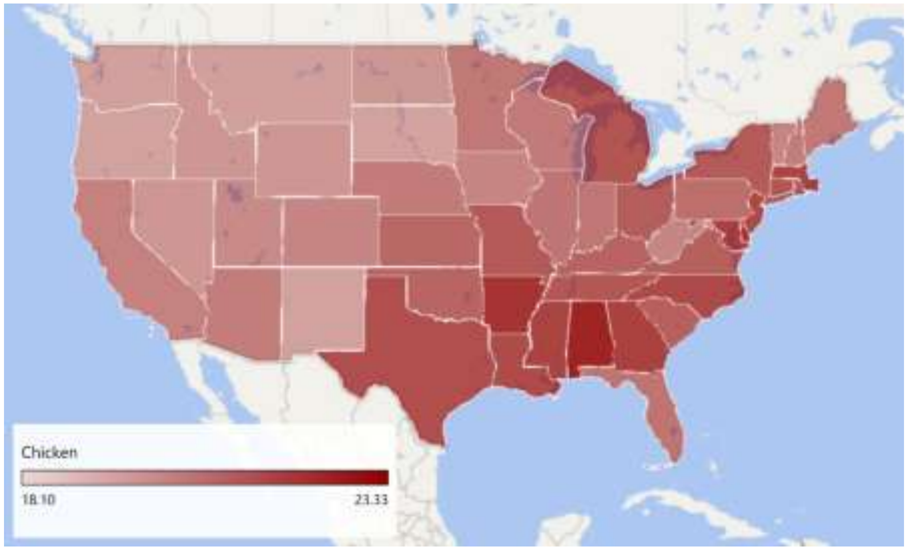

Queries for chicken recipes

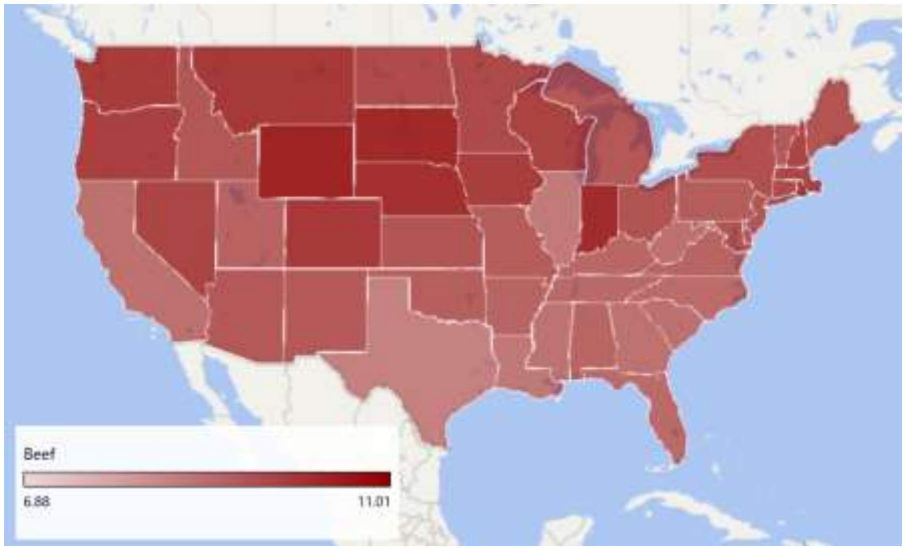

Queries for beef recipes

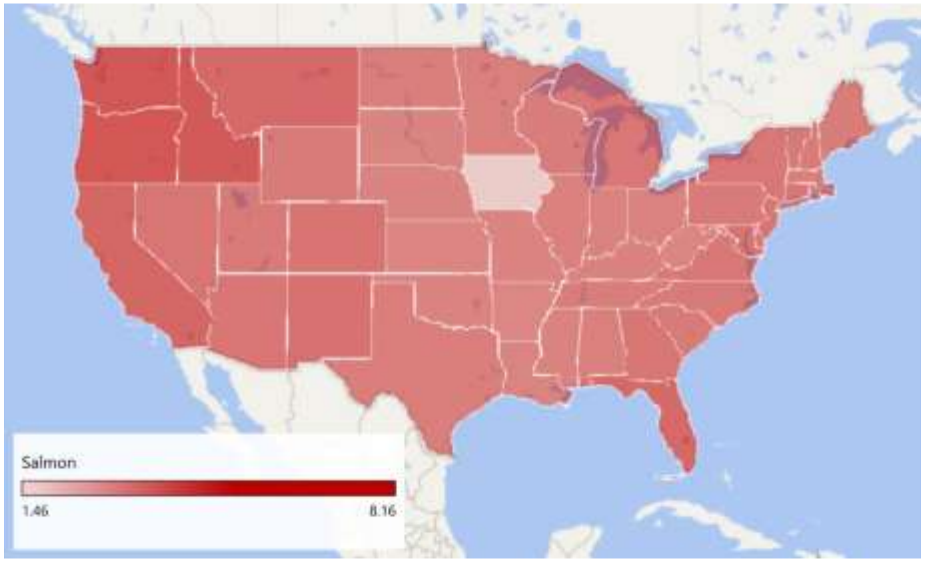

Queries for salmon recipes
