# Supplementary material for: Evidence From Web-Based Dietary Search Patterns to the Role of B12 Deficiency in Non-Specific Chronic Pain: A Large-Scale Observational Study
Source: J Med Internet Res. 2018 Jan 5;20(1):e4. doi: 10.2196/jmir.8667 (PMC5775484; doi:10.2196/jmir.8667)
Supplement: Multimedia Appendix 4 [file jmir_v20i1e4_app4.pdf]

Multimedia Appendix 4: Table 2 with regression coefficients for statistically significant interactions

	R^2I	CoB12	Beef	Chicken	Egg	Mackerel	Milk	Pig	Salmon	Shellfish	Trout	Tuna	Turkey	Tomato
**gabapentin**	3.39E-03	-0.219	-0.007	-0.006	-0.013	-0.003	-0.006	-0.010	-0.010	-0.015	-0.007	-0.013	-0.001	0.000
**tramadol**	3.35E-03	-0.207	-0.008	-0.007	-0.013	-0.003	-0.007	-0.012	-0.014	-0.016	-0.007	-0.015	-0.001	0.000
**neuropathy**	2.47E-03	-0.228	-0.007	-0.006	-0.011	-0.003	-0.006	-0.010	-0.009	-0.015	-0.007	-0.013	-0.001	0.000
**lyrica**	2.28E-03	-0.113	-0.004	-0.004	-0.007	-0.002	-0.004	-0.007	-0.006	-0.009	-0.004	-0.008	-0.001	
**omeprazole**	2.26E-03	-0.136	-0.004	-0.003	-0.007	-0.002	-0.003	-0.006	-0.005	-0.008	-0.003	-0.007	0.000	
**sertraline**	2.10E-03	-0.164	-0.003	-0.003	-0.005	-0.001	-0.003	-0.004	-0.003	-0.006	-0.002	-0.005	0.000	
**citalopram**	1.96E-03	-0.235	-0.003	-0.002	-0.004	-0.001	-0.002	-0.004	-0.003	-0.006	-0.002	-0.005	0.000	0.000
**oxycodone**	1.81E-03	-0.157	-0.005	-0.005	-0.009	-0.002	-0.004	-0.008	-0.006	-0.011	-0.004	-0.010	-0.001	
**duloxetine**	1.78E-03	-0.214	-0.002	-0.002	-0.004	-0.001	-0.002	-0.003	-0.003	-0.004	-0.002	-0.004	0.000	0.000
**trazodone**	1.77E-03	-0.182	-0.003	-0.003	-0.005	-0.001	-0.003	-0.005	-0.005	-0.007	-0.003	-0.006	0.000	
